# *In vitro* neutrophil migration is associated with inhaled corticosteroid treatment and serum cytokines in pediatric asthma

**DOI:** 10.3389/fphar.2022.1021317

**Published:** 2022-10-11

**Authors:** Solveig Lemmel, Markus Weckmann, Anna Wohlers, Adan Chari Jirmo, Ruth Grychtol, Isabell Ricklefs, Gyde Nissen, Anna Bachmann, Shantanu Singh, Juan Caicedo, Thomas Bahmer, Gesine Hansen, Erika Von Mutius, Klaus F. Rabe, Oliver Fuchs, Anna-Maria Dittrich, Bianca Schaub, Christine Happle, Anne E. Carpenter, Matthias Volkmar Kopp, Tim Becker, the ALLIANCE Study Group as part of the German Centre for Lung Research (DZL), Mustafa Abdo

**Affiliations:** ^1^ Department of Paediatric Pneumology and Allergology, University Children’s Hospital, Lübeck, Schleswig-Holstein, Germany; ^2^ Priority Research Area Chronic Lung Diseases Leibniz Lung Research Center Borstel, Epigenetics of Chronic Lung Disease, Großhansdorf, Germany; ^3^ Airway Research Center North, Member of the German Center of Lung Research (DZL), Lübeck, Germany; ^4^ Department of Pediatric Pneumology, Allergology and Neonatology, Hannover Medical School, Biomedical Research in Endstage and Obstructive Lung Disease Hannover (BREATH), Member of the German Center of Lung Research (DZL), Hannover, Germany; ^5^ Imaging Platform, Broad Institute of MIT and Harvard, Cambridge, CA, United States; ^6^ Department of Pneumology, LungenClinic Grosshansdorf, Airway Research Center North (ARCN), Member of the German Center for Lung Research (DZL), Grosshansdorf, Germany; ^7^ University Hospital Schleswig-Holstein Campus Kiel, Department for Internal Medicine I, Airway Research Center North (ARCN), German Center for Lung Research (DZL), Kiel, Germany; ^8^ University Children’s Hospital, Ludwig Maximilian’s University, German Research Center for Environmental Health (CPC-M), Member of the German Center of Lung Research (DZL), Munich, Germany; ^9^ Department of Paediatric Respiratory Medicine, Inselspital, University Children’s Hospital of Bern, University of Bern, Bern, Switzerland; ^10^ IAV GmbH, Gifhorn, Germany

**Keywords:** neutrophil granulocytes, migration, LTB4, fMLP, high-content image analysis, single-cell analysis

## Abstract

**Background:** Different asthma phenotypes are driven by molecular endotypes. A Th1-high phenotype is linked to severe, therapy-refractory asthma, subclinical infections and neutrophil inflammation. Previously, we found neutrophil granulocytes (NGs) from asthmatics exhibit decreased chemotaxis towards leukotriene B4 (LTB_4_), a chemoattractant involved in inflammation response. We hypothesized that this pattern is driven by asthma in general and aggravated in a Th1-high phenotype.

**Methods:** NGs from asthmatic nd healthy children were stimulated with 10 nM LTB_4_/100 nM N-formylmethionine-leucyl-phenylalanine and neutrophil migration was documented following our prior SiMA (simplified migration assay) workflow, capturing morphologic and dynamic parameters from single-cell tracking in the images. Demographic, clinical and serum cytokine data were determined in the ALLIANCE cohort.

**Results:** A reduced chemotactic response towards LTB_4_ was confirmed in asthmatic donors regardless of inhaled corticosteroid (ICS) treatment. By contrast, only NGs from ICS-treated asthmatic children migrate similarly to controls with the exception of Th1-high donors, whose NGs presented a reduced and less directed migration towards the chemokines. ICS-treated and Th1-high asthmatic donors present an altered surface receptor profile, which partly correlates with migration.

**Conclusions:** Neutrophil migration *in vitro* may be affected by ICS-therapy or a Th1-high phenotype. This may be explained by alteration of receptor expression and could be used as a tool to monitor asthma treatment.

## Introduction

Asthma is the most frequent chronic disease in children and highly prevalent in adults. Currently, about 300 million people are affected with a projected number of 400 million patients in 2025 (Croisant, 2014; Network, 2018). The diagnosis “asthma” is an umbrella term rather than a specific diagnosis with many possible causes and different phenotypic expressions (Kuruvilla et al., 2019), (Agache and Akdis, 2016). To clarify the situation, the terms asthma phenotype and asthma endotype have been coined: while an asthma phenotype is defined as a disease subtype associated with characteristic events, demographics, severity and therapy response, a specific asthma endotype is associated with characteristic pathophysiological mechanisms such as cellular and cytokine profiles (expression of Th1, Th2, Th9, Th17 at the mRNA and protein level) (Network, 2018; GINA Global Initiative for Asthma, 2019).

The most common endotype in children is the atopic/Th2 asthma subtype, characterized by high levels of Interleukin (IL-) 4, IL-5, or IL-13 and blood eosinophils, eosinophilic airway inflammation, and elevated exhaled nitrogen monoxide. This endotype is associated with early-onset allergic asthma, the most frequent phenotype amongst pediatric asthma patients. Early-onset allergic asthma includes all courses of the disease - from mild to severe (Boonpiyathad et al., 2019; Kuruvilla et al., 2019). Other endotypes may be Th2-low or mixed or characterized by Th1-related inflammation (definded by cytokines, such as IL-2 and interferon (IFN) ɣ), which is typical in patients with sputum neutrophilia, in smokers, or in older patients (Kuruvilla et al., 2019). But also in children with allergic and non-allergic severe asthma, Th1 signatures or mixed Th1/Th2 signatures have been described which are strongly associated with recent airway infections (Wisniewski et al., 2018).

In this context, little is known about the function and diagnostic relevance of neutrophil granulocytes (NGs). NGs are part of the innate immune system and represent the first line of defense against viral or bacterial airway infections, but also play a pivotal role in pathological lung tissue inflammation and airway damage in asthma (Pignatti et al., 2005). Chemotaxis towards pathogen-associated-molecular-patterns (PAMP) is mediated by a plethora of molecules including IL-8, leukotriene B4 (LTB_4_) and bacterial-derived peptide N-formylmethionine-leucyl-phenylalanine (fMLP) (de Oliveira et al., 2016). NGs from asthma patients have been shown to display reduced chemotactic speed towards fMLP compared to healthy donors (Sackmann et al., 2014). To assess neutrophil movement continuously during the migratory process on single cell level, we previously developed a simplified migration assay (SiMA) and found that NGs of asthmatic children were less responsive to an LTB_4_ gradient than those of healthy controls (Weckmann et al., 2017). In the current study, we aim to elucidate whether reduced NGs chemotaxis in response to LTB_4_ and fMLP was associated with features of Th1 inflammation with or without ICS therapy in children with asthma.

## Materials and methods

### Study population

From November 2017 to March 2019, blood samples from *n* = 75 asthmatic children and *n* = 13 healthy controls of the pediatric arm of the ALLIANCE cohort of the German Center for Lung Research [DZL, (Fuchs et al., 2018) clinicaltrials.gov (KIRA: NCT02496468)] were collected (all patients were 6 years or older). All in- and exclusion criteria are available in (Fuchs et al., 2018). Informed consent of all participants and/or their legal guardians was obtained, and ethical approval for all presented experiments was provided by the local ethics committee (Vote 12-215; 18.12.2012; ethics committee of the University of Lübeck).

Patients with asthma and healthy controls were invited yearly for a broad medical check-up (Fuchs et al., 2018). Besides acquisition of biomateriales (e.g., blood, stool, urine), study workups included lung function measurement, including spirometry, assessment of exhaled nitrogen monoxide (NO), whole-body plethysmography and multiple-breath washout. Interviews were used to record current medication, allergies and chronic diseases. General patient-relevant information (e.g., age, weight, height, gender and medication) was obtained at every visit.

A summary of the demographic parameters of asthmatic and healthy participants of this study is presented in Supplementary Table SA1.

### Clinical and demographical data

Continuous treatment with inhaled glucocorticoids (ICS) was inquired by questionnaire and categorized into healthy control, no, low, medium or high dose ICS treatment according to the GINA guidelines (GINA Global Initiative for Asthma, 2019). Medication that was taken on demand was assessed by questionnaire as well. Daily ICS doses were compared as fluticasone equivalent. To compensate for the resulting small numbers in some categories, the following classification was used:

- Control population (healthy, no asthma medication per definition, *n* = 13)
- Athmatics with no controller treatment (*n* = 31)
- Asthmatics taking low dose ICS controller treatment (<200ug fluticasone equivalent for children <12 years and <250 μg for children 12 years and older) *n* = 29 or low dose ICS treatment on demand (*n* = 5) and no additional controller medication
- Asthmatics taking moderate to high ICS dosis (containing asthmatics with a medium (*n* = 4; 200–400 µg fluticasone equivalent for children <12 years and 250–500 µg for children 12 years and older per day) and high (*n* = 6; <12 years = >400 µg/>12 years = >500 µg fluticasone equivalent per day) dose ICS treatment)

### Cell culture

Isolation of neutrophil granulocytes was performed after venous blood sampling, using ficoll density centrifugation and erythrocyte lysis as described in a Simplified Migration Assay for Analyzing Neutrophil Migration (SiMA). In addition to the SiMA-protocol, samples were stored a maximum of 12 h at 8°C between density centrifugation and lysis of erythrocytes. The final pellet was resuspended in 100 µl sterile human AB serum and stored at 8°C for 2 h, too. A cell count was performed using a Neubauer counting chamber (Marienfeld-Superior, Lauda Königshofen, Germany).

### Migration assay

Neutrophil migration was analyzed on a single cell level using the microfluidic migration assay, known as SiMA-workflow (Weckmann et al., 2017). Therefore, isolated NGs were mixed with 70μg/ml Fibronectin (FN; Sigma-Aldrich, St. Louis, Missouri, United States). Migratory behavior was observed towards the chemoattractants LTB_4_, (3.37 ng/ml; Abcam, Cambridge, England), IL8 (100 ng/ml; BD Biosciences, Franklin Lakes, New Jersey, United States) and fMLP (43.76 ng/ml; Abcam, Cambridge, England), utilizing µ-slides Chemotaxis 3D (IBIDI, Martinsried, Germany). Pictures were taken every 10 s for at least 30 min in a climate controlled room with a temperature of 20°C using the CytoSMART 2 System (CytoSMART, Einthoven, Netherlands). Image size was 1280 × 720 pixels with a pixel resolution of 0.96 µm per pixel stored in JPEG format.

Prior to analysis a quality check was performed excluding those experiments with a visible sidewards flow (cells with lateral trajectory, therefore skewing the gradient direction and prohibiting the establishment of chemotactic gradient), a too low cell number or pre-activated cells. This included cells that became stationary and did not migrate at all.

This quality control resulted in a different subset of migration experiments for each chemokine, compare Figure 2. To comprehensively assess proband specific NGs chemotaxis, at least 30 min of migration of at least *n* = 25 NGs were recorded. Supplementary Table SA3 gives an overview of the available experiments.

### Atopy

The atopy status was determined by questionnaire, being defined as the occurrence of allergic comorbidities or allergic sensitization with symptoms.

### Cytokine measurement

To measure blood cytokine levels, a bead ELISA Bio-Plex-Pro Human Cytokine 27-plex Assay was performed and measured with a Bio-Plex MAGPIX Multiplex Reader (both: BioRad, Hercules, United States), according to the manufacturer’s specifications.

### Chipcytometry based neutrophil granulocytes phenotyping

Chip cytometry was performed as previously described (Hennig et al., 2009; Jirmo et al., 2020a; Jirmo et al., 2020b). Briefly, approximately 2 × 105 isolated granulocytes were loaded and fixed on cell-adhesive microfluidic-chips according to company instructions (ZellSafe Chips, Zellkraftwerk GmbH, Leipzig, Germany). Neutrophils loaded on ZellSafe chips were then subsequently exposed to an iterative staining/bleaching cycle using ZellScannerONE (Zellkraftwerk GmbH, Leipzig, Germany). Phycoerythrin (PE) conjugated antibodies targeting BLT1, FPR1, CD62L, CD184, CD11b and CD16 (for specific clones, company and dilution details see Supplementary Table SA4) were used to characterize NGs. Data acquisition and analysis of fluorescence intensities was accomplished using the ZellExplorer App (Zellkraftwerk GmbH, Leipzig, Germany).

### Data analysis

Profiling of morphological dynamics Cell tracking data was processed in R following best practices and pipelines adapted from high throughput image analysis using the cytominer R package^1^.

### Image processing and cell tracking

**FIGURE 1:**
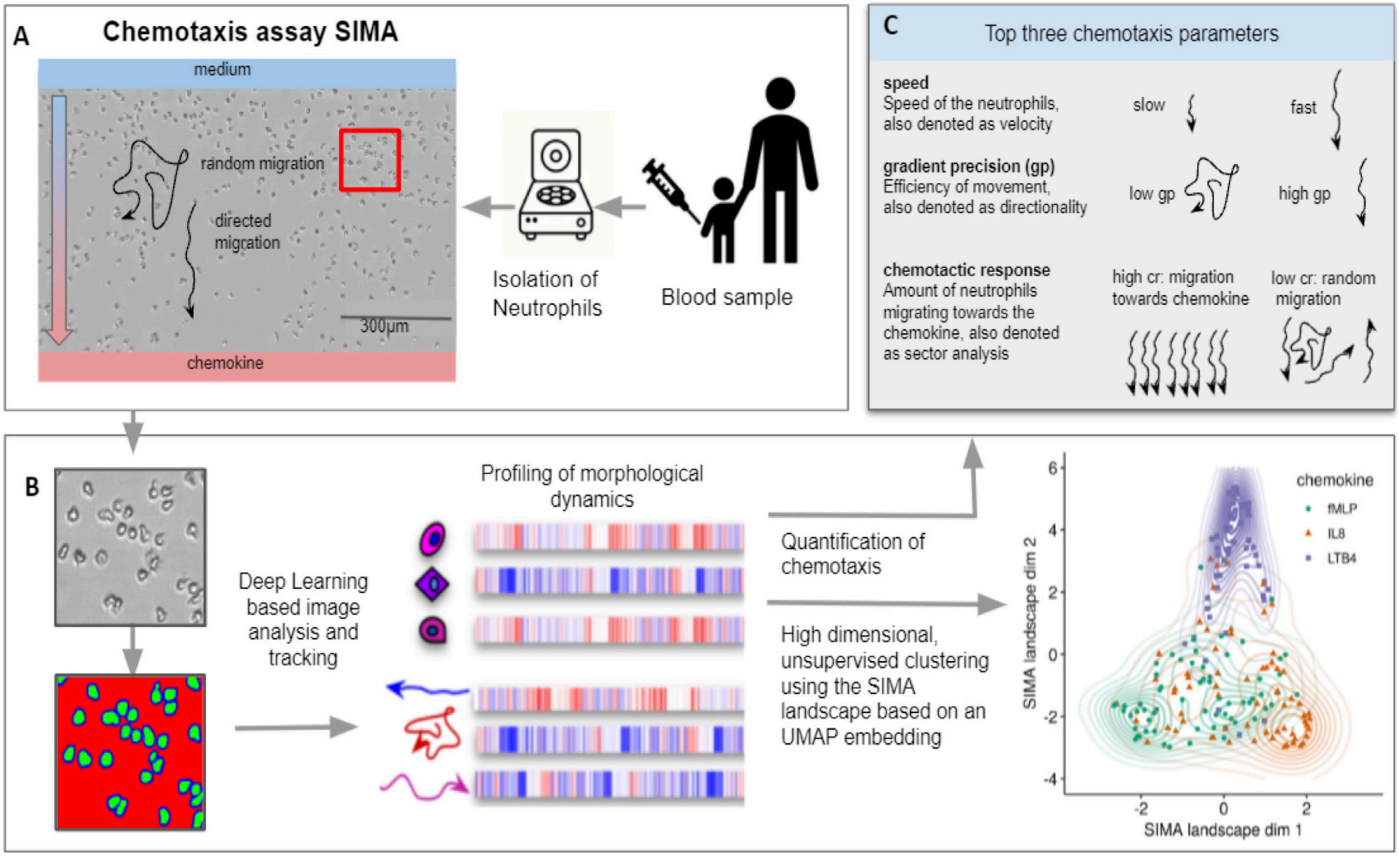
Pipeline of the SiMA chemotaxis analysis. **(A)** Neutrophils are isolated from a blood sample and chemotaxis is induced using a SiMA assay. For each patient, the migration towards IL8, fMLP and LTB_4_ is recorded using time lapse microscopy. **(B)** A Deep Learning based image segmentation (UNet model) is used to identify and track neutrophil granulocytes. For each experiment, single-cell and single-trajectory profiles are created using CellProfiler and MigrationmineR, describing each neutrophil with a total of 25 features (after feature selection). These features are aggregated on an experiment level and used for quantification of chemotaxis. **(C)** The most telling and interpretable migration parameters are speed, chemotactic response and the gradient precision. A multidimensional analysis is performed using the complete feature set, which is projected into a two dimensional SiMA landscape using the UMAP algorithm for dimension reduction.

To analyze NGs movement over time, neutrophils were identified by deep learning-based image segmentation. In detail, a three-class UNet (Ronneberger et al., 2015) was implemented^2^ and trained using 20 randomly chosen, manually labeled images. The UNet model predicts the probabilities for background, boundary of the NGs and foreground (NGs), compare Figure 1. The first 15 min of each 30-minute-long experiment were not evaluated as the gradient is not yet stable during this time. As a result, the cells were tracked and processed in a time window of 15 min length from 15 to 30 min after the experiment was started/the gradient was established.

Images and predictions were loaded into CellProfiler and cells were segmented, tracked over time and morphological information was extracted on single cell level. Morphological description parameters included texture, area, shape and intensity features as described by Becker et al. (Rapoport et al., 2011), Becker et al. (2018). To find a good set of morphological features, a selection was made to remove highly correlated features in single cell measurements (if two features had a pearson correlation >0.85, the one with the overall higher correlation was removed).

Next, dynamic features like speed, directionality and chemotactic response were extracted for each cell, which were denoted as trajectories, using the migrationmineR package^3^. For a short explanation of each parameter please see Figure 1.

To remove NGs that had not been tracked correctly, cells with a track length less than 50% of the observation time were removed. For the remaining trajectories, mean profiles were calculated describing the morphological dynamics of each experiment. These profiles were created by calculating the mean values of all dynamic and morphological features. The complete set included *n* = 25 features and is Supplementary Table SA5.

The data set was analyzed and four variables (gender, age, season and batch) were controlled for in a linear model. The corrected data set was normalized with respect to all experiments, i.e., the mean value of all features was set to 0 and the standard deviation to 1. The values for cytokine levels were log transformed, corrected for the variables age, gender, date of measurement and normalized with mean 0 and standard deviation 1.

Positive values describe a value larger than the mean of this feature across all experiments and across the chemokines IL8, fMLP, LTB_4_. Negative values describe a reduced value compared to the mean. For example for speed, a negative value does not imply a movement in reversed direction but a reduced speed that is lower than the mean speed of all neutrophils.

### Cluster analysis

All presented cluster analyses were performed as unsupervised clustering based on a “Uniform Manifold Approximation and Projection”-dimension reduction (McInnes et al., 2018). This clustering, using the extracted dynamic and morphologic migration parameters, conserves global connections and therefore allows to detect differences and similarities between single measurements. In the following, these global connections are displayed in the form of the SiMA landscape.

### Statistical analysis

Statistical analysis was performed using the statistical software R (v.3.6) and confirmed using JMP (JMP^®^, Version 14, SAS Institute Inc., Cary, NC, 1989-2019). Throughout the paper we use the following levels of significance: *p < 0.05, **p < 0.01, ***p < 0.001.

## Results

### Asthma reduces the chemotactic response of neutrophils

We ran migration experiments for a total of *n* = 75 asthmatic and *n* = 13 healthy donors using our live cell assay (Weckmann et al., 2017). The groups do not show significant differences in demographic or clinical variables except for exhaled NO and percentage of children with allergic symptoms, which was significantly elevated in the asthmatic donor population (*p* < 0.05) (Table 1).

**TABLE 1:** The asthmatic and healthy donor populations are very similar with no significant differences in demographic parameters, but asthma patients show a significantly increased exhaled NO and reported atopy (*p* < 0.05).

	Asthma (*n* = 75)	Control (*n* = 13)	Significance
Gender (male)	45 (60%)	5 (38.5%)	n.s.
Age	12.6 (±3.9)	13.1 (±3.4)	n.s.
BMI (body mass index)	21.6 (±6.5)	19.9 (±3.4)	n.s.
Reported Atopy	51 (68%)	3 (23.1%)	*p* = 0.0031
FEV1	104 (±13.3)	106.6 (±13.4)	n.s.
Exhaled NO	17.1 (±18.5)	9.8 (±11.3)	*p* = 0.0334
Eosinophils (%)	4.7 (±3.3)	3.5 (±3)	n.s.
Controlled asthma	36 (55.4%)		

**FIGURE 2:**
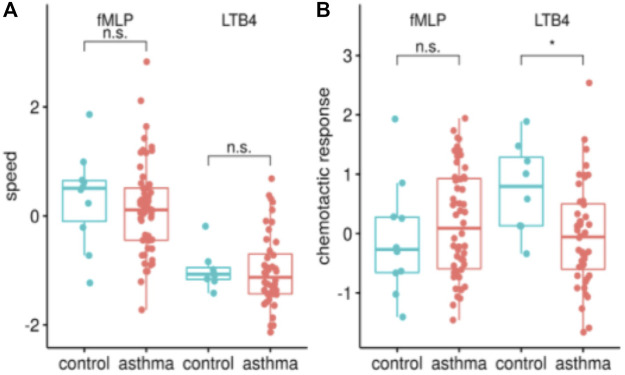
Asthma patients have a reduced chemotactic response towards LTB4, in terms of percentage of NGs moving as opposed to average speed. The analysis of the SiMA chemotaxis assays is characterized using the two most telling migration parameters, speed [z-value, in **(A)**] and chemotactic response [z-value, in **(B)**] for all migrations towards fMLP and LTB4. The distribution is shown for healthy controls (*n* = 10/8 fMLP/LTB4) and asthma patients (*n* = 55/45 for fMLP/LTB4). Migration parameters show a significantly reduced chemotactic response of asthma patients towards LTB_4_ (*p* < 0.05).

We found that migratory *speed* was not altered between patients and controls for either LTB_4_ (n asthma/control 45/8) nor fMLP (n asthma/control 55/10) (Figure 2A). However, the chemotactic response of NGs measured as the *percentage* of cells migrating towards the chemokine from asthmatic donors was significantly reduced towards LTB_4_ only (Figure 2B).

On average, 73% (z-value: 0.625 standard deviations from global mean) of NGs from healthy individuals migrated towards LTB_4_. In contrast, neutrophils from asthmatic donors only showed a directed migration towards the chemoattractant in 51% (z-value of 0.042) of trajectories. Interestingly, this was opposite to the migratory behavior towards fMLP. In addition to LTB_4_ and fMLP, IL8 was used as a chemoattractant. None of the effects described above occurred during IL8 induced chemotaxis (data not shown).

### Neutrophils of inhaled corticosteroid-treated asthmatic patients migrate similarly to healthy controls

**FIGURE 3:**
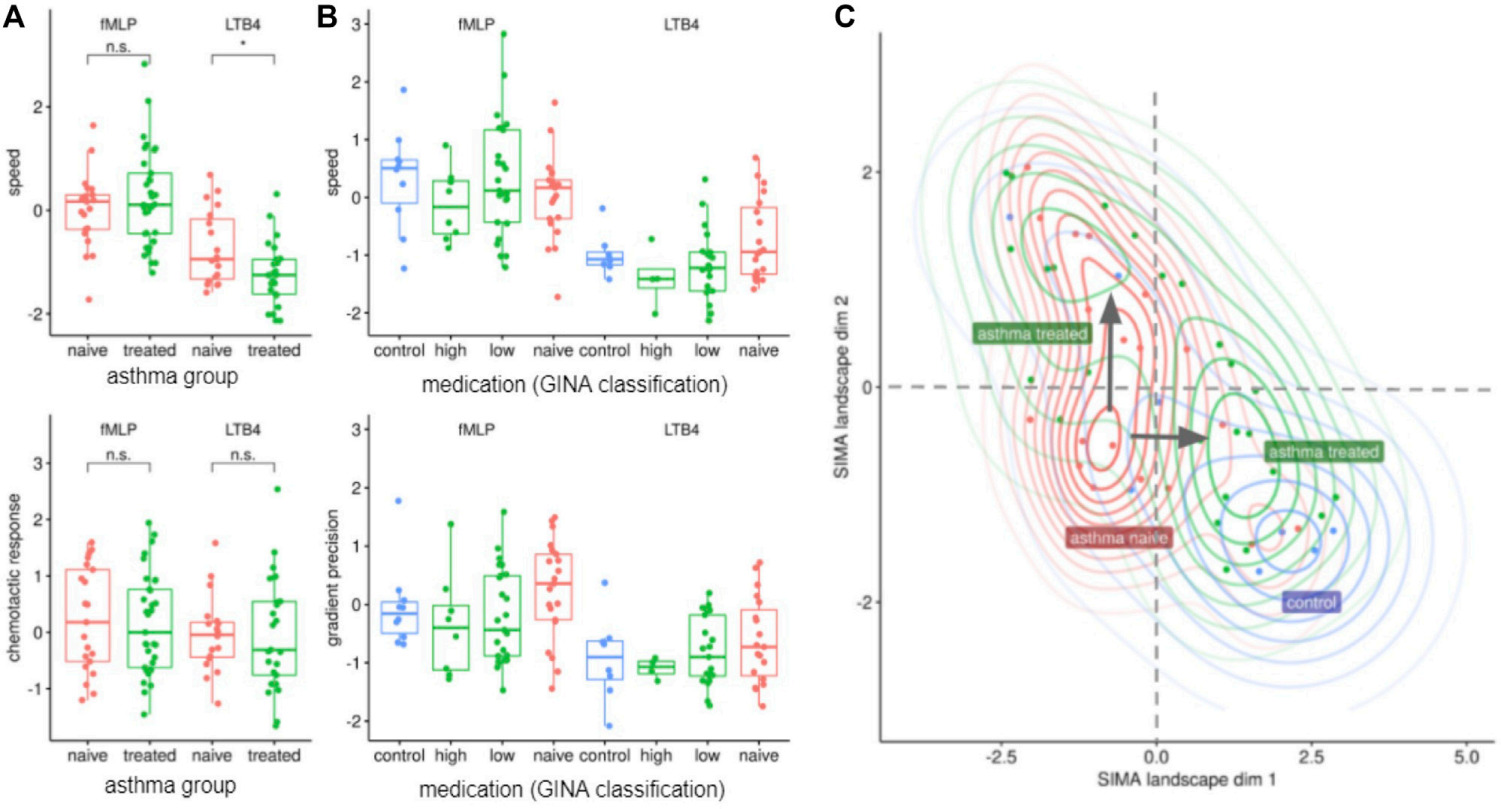
ICS-treated donors’ NGs show decreased speed of chemotaxis towards LTB4, but few other changes. **(A)** ICS treatment has a significant effect on the migration towards LTB_4_, reducing the speed (z-value) of neutrophils (*p*-value < 0.05, *t*-test), *n* = 26/19 for treated/naive). **(B)** Neutrophil migration parameters (z-values) grouped by ICS treatment doses. A patient specific dose was calculated as fluticasone equivalent based on the reported medication. The classes “control”, “high”, “low”, “naive” were defined according to GINA classifications and modified in two ways (to compensate for small n): First, class “low” includes patients with low daily doses and patients treated “as required”. Second, medium and high ICS dose were combined as “high”. **(C)** The SiMA landscape projects dynamic and morphological features into a two-dimensional embedding using the UMAP dimension reduction, which preserves similarities and distances between feature profiles from each patient. Asthma patients (red cluster lower left corner) and healthy controls (blue cluster lower right corner) have distinct migration profiles. When treated with inhaled corticosteroids (ICS), the profiles of Asthma patients form two main clusters: while most patients are located close to healthy controls (green cluster lower right corner), some patients show a pattern that is relatively distinct but close to a subset of from untreated asthma patients (green cluster upper left corner). Patients in this second cluster have increased vital capacity, decreased residual lung volume and a decreased total lung capacity. None of the determined cytokine profiles Th1, Th2, Th9 or Th17 are elevated in this cluster.

Next, we investigated how neutrophil migration compares *in vitro* in asthmatic patients with and without inhaled corticosteroid (ICS) treatment. Therefore, we analysed migratory behavior of asthmatic donors with reported ICS treatment (LTB_4_: *n* = 26; fMLP: *n* = 34) and those without ICS treatment (LTB_4_: *n* = 19; fMLP: *n* = 2); demographic and clinical parameters are presented in Supplementary Table SA1. There were no significant differences between age, gender, BMI or atopy between patients with and without ICS treatment. Unexpectedly, the speed towards LTB_4_ was significantly decreased in the group of ICS-treated asthmatics compared to untreated asthmatics (Figure 3A, *p* < 0.05). Still, neither the chemotactic response towards LTB_4_ nor migration parameters towards fMLP were significantly altered (Figures 3A,B). We explored this finding using additional information about each patient’s daily dose of ICS treatment; neither migration speed nor chemotactic response was linked to the daily dose of ICS treatment. A third parameter called gradient precision, which reports on the directionality of movement, showed a trend to increase in fMLP attracted NGs from ICS naive patients as compared to those with ICS treatment, but this did not reach significance (Figure 3B lower panel).

Unsupervised clustering based on migratory and morphological parameters identified four different clusters of patients: 1. a control cluster, 2. a cluster containing untreated asthmatics, 3. a cluster containing treated asthmatics and a 4th cluster containing a different subset of treated asthmatics (Figure 3C, see Materials and Methods for details).

The separability of control and ICS-untreated (ICS-naive) patients nicely confirmed our prior feature-specific analysis. This analysis also showed that *in vivo* ICS treatment resulted in asthmatics (asthmatics treated, cluster AT1, Figure 3C) being located in close proximity to healthy controls.

However, some treated asthmatics (cluster AT2) were located even further away from healthy control donors than the ICS naive group did. No significant differences between patients in cluster AT1 and AT2 were found, whether in lung function results or other clinical parameters (Supplementary Table SA1). In both groups of ICS-treated asthmatics, significantly less asthma control was reported as compared to ICS-naive asthmatics (naive: 84.6% vs. AT1: 34.4% vs. AT2: 37.5%, *p* < 0.01), indicating that the ICS-treated asthmatics include patients with higher disease severity.

### Neutrophils of Th1-high asthmatics show a reduced and randomized migration

**FIGURE 4:**
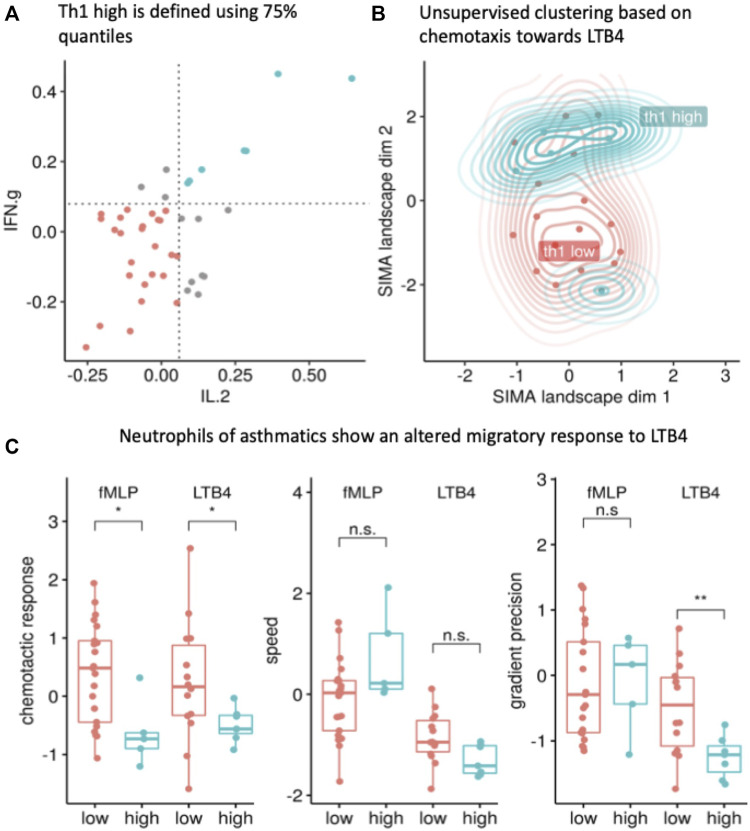
The Th1-high asthma phenotype (blue) corresponds to a unique chemotaxis pattern. **(A)** The Th1-high phenotype (marked in blue) is defined as a patient with interleukin 2 (IL2) and interferon gamma (IFNɣ) levels above the >75% quantile (quantiles are shown as dotted line; each dot represents one patient and only patients with valid readouts for IL2 and IFNɣ were included; Th1-low donors are presented in red). **(B)** The morphological dynamics are projected into the SiMA landscape and show a cluster of Th1-high asthma patients. The 2D embedding was calculated using a UMAP projection of all morphological and dynamic features based on chemotaxis experiments towards LTB_4_ with IL2 and IFNɣ measurements (*n* = 20 with 13 Th1-low and 7 Th1-high). **(C)** The migratory response (z-value) of Th1-high NGs in a LTB_4_ gradient shows a significant difference compared to neutrophils isolated from Th1-low donors (reduced chemotactic response (z-value), *p* < 0.01, *t*-test) and reduced gradient precision (z-value, *p* < 0.01, *t*-test). The migration towards fMLP shows a similar effect for the chemotactic response (z-value, *p* < 0.05, *t*-test).

The effects between ICS naïve and treated patients for the dynamic parameters were largely insignificant, hence we did not choose to adjust for them. In a next, we investigated asthma phenotypes based on a Th1 serum cytokine signature. Asthmatics with elevated levels of both cytokines IL-2 and IFNɣ (above the 75% quantile, Figure 4A), were defined to be “Th1-high” (*n* = 7) (Table 2). Asthmatic donors were labeled “Th1-low” (*n* = 30), if none of the cytokines were elevated. The remaining intermediate asthmatic donors (*n* = 38) were omitted from this analysis. No control donor presented with a Th1-high cytokine profile; these were also ignored in this analysis.

**TABLE 2:** Demographics of the Th1-high/low donor population. The Th1-high phenotype is characterised by significantly increased interleukin 6 (IL6), IL1b, TNFa and G-CSF level (**p < 0.01, ***p < 0.001), and a significant difference in BMI but no other patient demographics.

	Th1-low (*n* = 30)	Th1-high (*n* = 7)	*p*-value
Gender (male)	16 (53%)	4 (57%)	n.s.
Age	12 (±3.9)	10.6 (±3.9)	n.s.
BMI	20.9 (±5.4)	16.7 (±1)	*p* = 0.0003
ICS treatment (%)	20 (67%)	5 (71%)	n.s.
Reported atopy (%)	21 (70%)	3 (43%)	n.s.
Exh. NO	22.1 (±23.1)	15.6 (±17.3)	n.s.
Tiffaneau Index	98.9 (±8.4)	105.4 (±9.1)	n.s.
Eosinophils (%)	5.2 (±3.7)	5.4 (±2.9)	n.s.
IL-6	−0.032 (±0.153)	0.248 (±0.174)	*p* = 0.0041
IL1β	−0.049 (±0.126)	0.229 (±0.264)	*p* = 0.0314
TNFɑ	−0.017 (±0.122)	0.3 (±0.169)	*p* = 0.0018
G-CSF	−0.0527 (±0.125)	0.186 (±0.214)	*p* = 0.0387
Controlled asthma	15 (58%)	3 (43%)	n.s.

Th1-high asthmatics displayed a significantly (*p* < 0.0003) lower BMI than Th1-low donors but there were no other significant differences in the demographic data (Table 2). The majority of Th1-high donors were non-atopic and treated more often with ICS as compared to Th1-low donors. When examining the measured cytokine levels in both groups, Th1-high asthmatics presented significantly elevated levels of IL1β, IL6, TNFɑ and G-CSF (Table 2). Th1-high patients showed a trend towards less controlled asthma, however due to the small numbers, significance was not reached (Table 2). Of note, out of the seven Th1-high patients, three were classified as Th2-high patients (Supplementary Table SA2).

Again, we performed unsupervised UMAP clustering based on morphologic and dynamic migration parameters (Figure 4B). Here, two different clusters were detectable. While Th1-low donors were associated in one loose cluster, a second, very dense cluster containing almost all Th1-high donors was obvious. The only exception was one outlier with a markedly elevated level of exhaled NO (exh. NO > 90ppb) and overall, very dissimilar migration patterns as compared to the rest of the Th1 cluster. This outlier was removed from further analysis.

In a comparison based on specific migratory parameters, Th1-high donors were characterized by significantly reduced chemotactic responses (percent of cells) towards LTB_4_ and fMLP (Figure 4C left panel, *p* < 0.05). Speed did not differ significantly in any chemotactic condition Figure 4C panel in the middle). Additionally, gradient precision towards LTB_4_ also was significantly reduced, indicating that compared to Th1-low donors, fewer neutrophils from Th1-high donors migrated towards the stimulus while also being slower and less directed (Figure 4C, right panel).

### Some asthmatic subpopulations present altered surface expression of receptors, which partially correlates with migratory behavior

**FIGURE 5:**
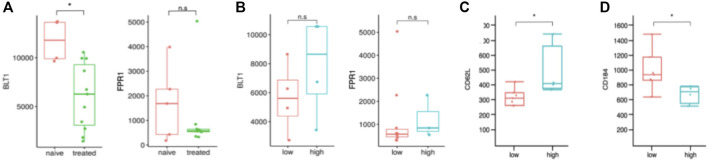
Surface receptor expressions of asthmatic patients could explain the altered migration behavior. **(A)** Comparison of BLT1 and FPR1 expression on neutrophils of corticosteroid naive and treated patients. Fluorescence units arbitrary on y-axis; BLT1: *n* = 4 naive and *n* = 10 treated. FPR1: *n* = 5 naive and *n* = 7 treated. n.s. not significant, *p < 0.05, Wilcoxon. **(B)** Comparison of BLT1 and FPR1 expression on neutrophils of patients with Th1 high and low profiles. No significant difference was detected. Fluorescence units arbitrary on y-axis; BLT1: *n* = 4 low and *n* = 4 high. FPR1: *n* = 8 low and *n* = 3 high. n.s., not significant. Comparison of CD62L **(C)** and CD184 **(D)** expression on neutrophils of patients with Th1 high and low profiles. Fluorescence units arbitrary on y-axis; CD62L: *n* = 4 high and *n* = 8 low. CD184: *n* = 4 high and *n* = 7 low. *p < 0.05, Wilcoxon.

Next, we were interested in the cause of the altered migratory behavior. Therefore, we analyzed the expression level of the chemokine receptors for LTB_4_ (BLT1; *n* = 14) and fMLP (FPR1; *n* = 12) for a subgroup of our patients. We observed significantly less BLT1 on neutrophils of patients treated with ICS as compared to untreated asthmatics (Figure 5A, left panel, *p* < 0.05).

We did not detect a changed expression of FPR1 between treated and untreated asthmatics (Figure 5A, right panel). This fits our observation that the migratory speed of neutrophils attracted to fMLP is similar between both groups (Figure 4C).

Furthermore, we examined Th1-high vs. Th1-low asthma patients but did not detect a significant difference in receptor expression between these groups, neither in BLT1 nor FPR1 expression (Figure 5B, left and right panel). Nevertheless, neutrophils of Th1-high patients expressed significantly more CD62L (*p* < 0.05, L-selectin, Figure 5C) and less CD184 (*p* < 0.05, CXCR4, Figure 5D).

## Discussion

We present here the comprehensive migration analysis on single cell level of NGs from asthmatic children and healthy controls thus far. NGs from children with asthma display impaired *in vitro* chemotactic responses towards LTB_4_. However, ICS treatment largely had no significant effects on dynamic migration parameters. A less directed and reduced chemotactic response towards LTB_4_ was observed in Th1-high asthmatic children.

Previously, we reported a reduced *in vitro* chemotaxis of NGs towards LTB_4_ for asthmatic patients (Weckmann et al., 2017). Here, we confirm this phenomenon in a larger cohort and show a reduced percentage of directed trajectories when stimulated by LTB_4_ (73.4% vs. 51.68%) but not for fMLP or IL8. The latter did not show any difference between disease status, treatment or phenotype (data not shown). The migration effect is preserved, confirming that peripheral neutrophils from asthmatic children respond less to LTB_4_ than healthy controls.

We did not replicate the observation by Sackman and colleagues that asthmatic neutrophils migrate slower in an fMLP gradient, regardless of ICS treatment (Sackmann et al., 2014). Several differences (fibronectin vs. p-selectin coating; density gradient centrifugation vs. blood drop) between the two microfluidic platforms exist, which may account for the lack of comparability.

In order to identify the asthmatic phenotype associated with this migratory behavior we investigated the difference between ICS-treated and non-treated children. We previously described a reversal of the chemotactic response *in vitro* when NGs were incubated with prednisolone. However, in this study we analyzed the *in vitro* chemotaxis of NGs from ICS treated asthmatic children compared to untreated children and healthy controls. We found a reduced migration speed (LTB_4_) in the cohort of ICS-treated children, while we could not detect a significant change of the chemotactic response (either fMLP or LTB_4_) or gradient precision even after stratifying for ICS dosage. (Weckmann et al., 2017) An unsupervised analysis using the high dimensional morphologic and dynamic features from our tracking data, revealed a 2-dimensional SiMA landscape, in which ICS-treated asthmatics and control patients showed a similar chemotaxis pattern. The only exception is a small fraction of ICS-treated patients (cluster AT2 in Figure 4C), very far from healthy controls and from the majority of the treated patients as well. We found no significant difference in clinical of demographical data defining this cluster. Further research is warranted to better understand the patients in this special migratory cluster and the relevance of the altered neutrophil migration.

Next, we used the serum cytokine levels to define immunological “endotypes” to characterize their migration pattern. We identified patients with elevated Th1 cytokines (IL-2 and IFNɣ) whose NGs are characterized by a below average chemotactic response towards LTB_4_. Further, the SiMA landscape projection clearly separated the Th1-high and Th1-low patients into two clusters. We did not find significant differences in chemotactic responses in Th2, Th9 or Th17 (Supplementary Figure SA1).

In order to identify underlying causes for the altered migratory behavior, we analysed NGs surface receptor expression in our asthma patients. We found less BLT1 protein expressed on the surface of neutrophils from ICS-treated asthmatic patients that migrate slower towards LTB_4_. Qasaimeh et al. subjected NGs to different levels of IL-8 gradients and found that at about 90% of the maximal IL-8 concentrations, the chemotactic speed reduced significantly (Qasaimeh et al., 2018). The authors suggested a saturation of the IL-8 receptor to be causative for this behavior. Lower BLT1 levels in our assay would result in earlier termination of migration due to receptor saturation in our gradient and an overall reduced migratory speed. IL-8 and LTB_4_ belong to so-called “intermediate chemokines” and migration response is distinct as compared to end-target chemoattractants such as fMLP (reviewed in (Sadik et al., 2011)). Petrie Aronin et al. showed that whilst intermediate chemokines require an ever-increasing concentration to stably recruit neutrophils, for end-target chemoattractants a stable gradient suffices (Petrie Aronin et al., 2017). In particular, the authors argue that in devices like the ones used in our study (3D Ibidi slide) the gradient is not fully established in the initial phase, so intermediate chemokine concentration follows an ever-increasing pattern until a steady-state level is achieved. Any migration measurements under these conditions would eliminate differences between IL-8/LTB_4_ and fMLP.

In our analysis, we only used images starting from 15min into the migration experiment until the end (after 30 min). Also, we did not see any of the LTB_4_ migration effects to occur in IL-8 recruited neutrophils, which suggests that the effect of LTB_4_ is chemokine-specific at least in direct comparison with IL-8. However, levels of chemokine receptor expression (FPR1 and BLT1) did not explain the difference in the chemotactic response. This may point towards an alternative hypothesis, in which NGs are primed by the existing milieu and therefore respond differently to chemotactic cues. This may unfold in two distinct scenarios:

A. Peripheral neutrophils exposed to chemokines in serum are desensitized and less responsive towards those (and other) chemokines. It is known that CXCR1 ligation leads to a reduced BLT1-induced chemotaxis (Tarlowe et al., 2003). In our Th1-high patients IL-8 is significantly increased in serum, but no difference in migration speed or chemotactic response towards IL-8 is observed (data not shown). In addition, we did not see a decrease of BLT1 in neutrophils from Th1-high patients.
B. Alternately, the neutrophil population is primed or distinctly different in its composition. In our study, the reduced chemotactic response of peripheral neutrophils from Th1 high asthmatics was associated with decreased CD184 (CXCR4) levels. Numbers of CXCR4 high neutrophils are significantly reduced in peripheral blood and the bone marrow after intraperitoneal injection of LPS in mice. Recently, Uhl et al. showed that elevated CXCR4 expression is found on “aged” neutrophils, which are more likely to respond to LPS injection in mice and lead to increased levels of CXCR4 high neutrophils in liver, kidney and lung (Uhl et al., 2016). Furthermore, approximately 50% of all NGs in the lung belonged to the CXCR4 high (“aged”) phenotype. This is supported by earlier work from Yamada et al. Yamada et al. (2011) who identified CXCR4 expression to be increased on extravascular NGs after endotoxin-induced lung injury. Nasal LPS installation led to a significant increase of neutrophil influx in their mouse model characterized by higher CXCR4 expression on neutrophils. Blocking CXCR4 or CXCL12 (the only known ligand for CXCR4) resulted in a significant decrease in neutrophil influx in LPS induced lung injury. CXCL12 has been reported to be increased in serum and broncho-alveolar lavage in asthmatics and is produced by the airway epithelium during asthma exacerbation (Daubeuf et al., 2013; Wang et al., 2015). This may suggest that in asthmatic patients with a Th1-high signature more “aged” neutrophils are recruited as a consequence of a recent infection or lack of resolution of inflammation, whilst “younger” NGs are found in circulation. These younger neutrophils may respond differently to LTB_4_. Further experiments with CXCR4 high/low neutrophils and LTB_4_ induced chemotaxis may help to unravel this intricate relationship.

Evidence for a complex interplay of recent infections and Th1 “endotype” characteristics in asthmatic children is presented by Wisniewski and colleagues. In their study, current or recent viral or bacterial infection were associated with persistently increased Th1 cytokines and Th1 lymphocytes in broncho-alveolar lavage fluid (BALF) (Wisniewski et al., 2018). Additionally, Grunwell et al. demonstrated that BALF from severe, neutrophil high, asthmatic children stimulated peripheral neutrophils to increase CD62L (L-selectin expression) and become more pro-inflammatory (Grunwell et al., 2019). Interestingly, we found CD62L to be significantly increased in our Th1-high asthma NGs population. On the other hand, a lack of proresolving mediators such as lipoxin A4 (LXA_4_), may result in differentially primed neutrophils. LXA_4_ was decreased in peripheral blood, sputum and BALF of severe asthma patients (Ricklefs et al., 2017). LXA_4_ correlated inversely with the number of airway neutrophils especially in severe asthma patients (Ricklefs et al., 2017). Th1-high asthma is more prevalent in the group of severe asthma patients. Whether unresolved inflammation or recent infections remains to be elucidated but may support an endotype-related priming environment supportive of our alternative hypothesis (see hypothesis B in previous paragraph).

Our study has several limitations. First, the overall number of individuals in our migration cohort is relatively small. This poses problems especially when smaller subgroups are investigated and compared. Although we see an ICS dose-associated difference in NGs migration speed, we lack statistical power to confirm this difference. In a future study, a double-blinded, randomized, placebo-controlled cross-over design could help to disentangle possible dose relationships of ICS on *ex vivo* neutrophil migration. Furthermore, overall numbers of patients in our SiMA landscape analysis of ICS use are too small. We also did not succeed in recruiting sufficient numbers of healthy controls in our second batch for our cytochip analysis to compare BLT1, CXCR4, CD62L and FRP1 expressions from asthmatics and controls. As we observe the strongest loss of chemotactic response in Th1-high vs. -low asthma patients, the lack of a direct comparison to controls is however less critical. In addition, although the overall effect of reduced chemotactic response appears to be preserved, the actual percentages differ from our previous results. In our initial setup we used a single video-microscope setup (EVOS) as compared to a multiple video-microscope array (CytoSmart) in our current study. This increased our throughput and data generation at the expense of exact replication of our previous results. Furthermore, in this study we continuously recruited over several years as compared to a single season in our previous study. As it is known that cytokine levels differ significantly in between seasons this might influence comparability (Weckmann et al., 2021). Another limitation is that despite the elevated levels of Th1 cytokines, we did not investigate severe childhood asthmatics. Lung function was relatively normal in our asthmatic children and even our Th1-high patients did not show a pronounced reduction of FEV1% or the Tiffeneau index. This is in contrast to the studies by Grunwell and Wisniewski, which analysed more severe childhood asthma cases. Therefore, one needs to be careful to extrapolate our results to more severe asthma without further experiments in such individuals.

Taken together, this study identifies neutrophil migration to be associated with asthma therapy (ICS) and related to a “Th1” endotype in asthma. NGs isolated from peripheral blood retained their priming or programming as such, which enabled clustering and identification of subtypes of patients by morphologic and dynamic migratory parameters. This suggests that the underlying asthma endotype/theratype has direct consequences for granulocyte effector functions. Analysis of neutrophil migration with the SiMA protocol paired with neutrophil receptor analysis may be a valuable strategy to monitor effective treatment in children with asthma.

## ALLIANCE Study Group

**Mustafa Abdo**, LungenClinic Grosshansdorf GmbH, Grosshansdorf, Germany, and Airway Research Center North (ARCN), Germany; German Center for Lung Research (DZL), **Miguel Alcazar**, University of Cologne, Faculty of Medicine and University Hospital Cologne, Translational Experimental Pediatrics - Experimental Pulmonology, Department of Pediatric and Adolescent Medicine, Germany, University of Cologne, Faculty of Medicine and University Hospital Cologne, Center for Molecular Medicine Cologne (CMMC), Germany, Excellence Cluster on Stress Responses in Aging-associated Diseases (CECAD), University of Cologne, Faculty of Medicine and University Hospital Cologne Cologne, Germany, and Institute for Lung Health, University of Giessen and Marburg Lung Centre (UGMLC), Member of the German Centre for Lung Research (DZL), Gießen, Germany, **Thomas Bahmer**, LungenClinic Grosshansdorf GmbH, Grosshansdorf, Germany, and Airway Research Center North (ARCN), Germany; German Center for Lung Research (DZL), **Mira Berbig**, Department of Paediatric Allergology, Dr von Hauner Children’s Hospital, Ludwig Maximilians University, Munich, Germany, and Comprehensive Pneumology Center, Munich (CPC-M), Germany; German Center for Lung Research (DZL), Heike Biller, LungenClinic Grosshansdorf GmbH, Grosshansdorf, Germany, and Airway Research Center North (ARCN), Germany; German Center for Lung Research (DZL), **Xenia Bovermann**, University Children’s Hospital, Luebeck, Germany, and Airway Research Center North (ARCN), Germany; German Center for Lung Research (DZL), **Folke Brinkmann**, Department of Paediatric Pneumology, Allergology and Neonatology, Hannover Medical School, Hannover, Germany, and Biomedical Research in Endstage and Obstructive Lung Disease Hannover (BREATH), Germany; German Center for Lung Research (DZL), and Department of Paediatric Pneumology, University Children’s Hospital, Ruhr-University Bochum, Bochum, Germany, **Mifflin-Rae Calveron**, Hannover Medical School, Hannover, Germany, and Biomedical Research in Endstage and Obstructive Lung Disease Hannover (BREATH), Germany; German Center for Lung Research (DZL), **Adan Chari Jirmo**, Department of Paediatric Pneumology, Allergology and Neonatology, Hannover Medical School, Hannover, Germany, and Biomedical Research in Endstage and Obstructive Lung Disease Hannover (BREATH), Germany; German Center for Lung Research (DZL), **David S. DeLuca**, Hannover Medical School, Hannover, Germany, and Biomedical Research in Endstage and Obstructive Lung Disease Hannover (BREATH), Germany; German Center for Lung Research (DZL), **Gesa Diekmann**, University Children’s Hospital, Luebeck, Germany, and Airway Research Center North (ARCN), Germany; German Center for Lung Research (DZL), **Anna-Maria Dittrich**, Department of Paediatric Pneumology, Allergology and Neonatology, Hannover Medical School, Hannover, Germany, and Biomedical Research in Endstage and Obstructive Lung Disease Hannover (BREATH), Germany; German Center for Lung Research (DZL), **Christian Dopfer**, Department of Paediatric Pneumology, Allergology and Neonatology, Hannover Medical School, Hannover, Germany, and Biomedical Research in Endstage and Obstructive Lung Disease Hannover (BREATH), Germany; German Center for Lung Research (DZL), **Markus Ege**, Department of Paediatric Allergology, Dr von Hauner Children’s Hospital, Ludwig Maximilians University, Munich, Germany, and Comprehensive Pneumology Center, Munich (CPC-M), Germany; German Center for Lung Research (DZL), **Svenja Foth**, University Children’s Hospital Marburg, University of Marburg, Germany, and University of Giessen Marburg Lung Center (UGMLC); Member of the German Center for Lung Research, **Oliver Fuchs**, Department of Paediatric Allergology, Dr von Hauner Children’s Hospital, Ludwig Maximilians University, Munich, Germany, and Comprehensive Pneumology Center, Munich (CPC-M), Germany; German Center for Lung Research (DZL), and Department of Paediatric Respiratory Medicine, Inselspital, University Children’s Hospital of Bern, University of Bern, Bern, Switzerland, **Svenja Gaedcke**, Hannover Medical School, Hannover, Germany, and Biomedical Research in Endstage and Obstructive Lung Disease Hannover (BREATH), Germany; German Center for Lung Research (DZL), **Karoline I. Gaede**, Research Center Borstel—Medical Clinic, Borstel, Germany, and Airway Research Center North (ARCN), Germany; German Center for Lung Research (DZL), **Ruth Grychtol**, Department of Paediatric Pneumology, Allergology and Neonatology, Hannover Medical School, Hannover, Germany, and Biomedical Research in Endstage and Obstructive Lung Disease Hannover (BREATH), Germany; German Center for Lung Research (DZL), **Anika Habener**, Department of Paediatric Pneumology, Allergology and Neonatology, Hannover Medical School, Hannover, Germany, and Biomedical Research in Endstage and Obstructive Lung Disease Hannover (BREATH), Germany; German Center for Lung Research (DZL), **Gesine Hansen**, Department of Paediatric Pneumology, Allergology and Neonatology, Hannover Medical School, Hannover, Germany, and Biomedical Research in Endstage and Obstructive Lung Disease Hannover (BREATH), Germany; German Center for Lung Research (DZL), **Christine Happle**, Department of Paediatric Pneumology, Allergology and Neonatology, Hannover Medical School, Hannover, Germany, and Biomedical Research in Endstage and Obstructive Lung Disease Hannover (BREATH), Germany; German Center for Lung Research (DZL), **Christian Herzmann**, Research Center Borstel – Medical Clinic, Borstel, Germany, and Airway Research Center North (ARCN), Germany; German Center for Lung Research (DZL), **Alexander Hose**, Department of Paediatric Allergology, Dr von Hauner Children’s Hospital, Ludwig Maximilians University, Munich, Germany, and Comprehensive Pneumology Center, Munich (CPC-M), Germany; German Center for Lung Research (DZL), **Sabina Illi**, Institut für Asthma- und Allergieprävention (IAP), Helmholtz Zentrum Munich, Deutsches Forschungszentrum für Gesundheit und Umwelt (GmbH), Munich, Germany, **Anne-Marie Kirsten**, Pulmonary Research Institute at LungenClinic Grosshansdorf, Grosshansdorf, Germany, and Airway Research Center North (ARCN), Germany; German Center for Lung Research (DZL), **Naschla Kohistani-Greif**, Department of Paediatric Allergology, Dr von Hauner Children’s Hospital, Ludwig Maximilians University, Munich, Germany, and Comprehensive Pneumology Center, Munich (CPC-M), Germany; German Center for Lung Research (DZL), **Inke R. König**, Institute for Medical Biometry and Statistics, University Luebeck, University Medical Centre Schleswig-Holstein, Campus Luebeck, Germany, and Airway Research Center North (ARCN), Germany; German Center for Lung Research (DZL), **Silke Van Koningsbruggen-Rietschel**, University of Cologne, Faculty of Medicine and University Hospital Cologne, Department of Pediatrics, Cologne, Germany, **Matthias V. Kopp**, University Children’s Hospital, Luebeck, Germany, and Airway Research Center North (ARCN), Germany; German Center for Lung Research (DZL), **Johanna Kurz**, Department of Paediatric Allergology, Dr von Hauner Children’s Hospital, Ludwig Maximilians University, Munich, Germany, and Comprehensive Pneumology Center, Munich (CPC-M), Germany; German Center for Lung Research (DZL), and Department of Paediatric Respiratory Medicine, Inselspital, University Children’s Hospital of Bern, University of Bern, Bern, Switzerland, **Katja Landgraf-Rauf**, Department of Paediatric Allergology, Dr von Hauner Children’s Hospital, Ludwig Maximilians University, Munich, Germany, and Comprehensive Pneumology Center, Munich (CPC-M), Germany; German Center for Lung Research (DZL), **Kristina Laubhahn**, Department of Paediatric Allergology, Dr von Hauner Children’s Hospital, Ludwig Maximilians University, Munich, Germany, and Comprehensive Pneumology Center, Munich (CPC-M), Germany; German Center for Lung Research (DZL), **Lena Liboschik**, University Children’s Hospital, Luebeck, Germany, and Airway Research Center North (ARCN), Germany; German Center for Lung Research (DZL), **Claudia Liebl**, Department of Paediatric Allergology, Dr von Hauner Children’s Hospital, Ludwig Maximilians University, Munich, Germany, and Comprehensive Pneumology Center, Munich (CPC-M), Germany; German Center for Lung Research (DZL), **Berrit Liselotte Husstedt**, University Children’s Hospital, Luebeck, Germany, and Airway Research Center North (ARCN), Germany; German Center for Lung Research (DZL), **Bin Liu**, Hannover Medical School, Hannover, Germany, and Biomedical Research in Endstage and Obstructive Lung Disease Hannover (BREATH), Germany; German Center for Lung Research (DZL), **Nicole Maison**, Department of Paediatric Allergology, Dr von Hauner Children’s Hospital, Ludwig Maximilians University, Munich, Germany, and Comprehensive Pneumology Center, Munich (CPC-M), Germany; German Center for Lung Research (DZL), and Institut für Asthma- und Allergieprävention (IAP), Helmholtz Zentrum Munich, Deutsches Forschungszentrum für Gesundheit und Umwelt (GmbH), Munich, Germany, **Aydin Malik**, Department of Paediatric Pneumology, Allergology and Neonatology, Hannover Medical School, Hannover, Germany, and Biomedical Research in Endstage and Obstructive Lung Disease Hannover (BREATH), Germany; German Center for Lung Research (DZL), **Carola Marzi**, Institut für Asthma- und Allergieprävention (IAP), Helmholtz Zentrum Munich, Deutsches Forschungszentrum für Gesundheit und Umwelt (GmbH), Munich, Germany, **Meike Meyer**, University of Cologne, Faculty of Medicine and University Hospital Cologne, Department of Pediatrics, Cologne, Germany, **Erika Von Mutius**, Department of Paediatric Allergology, Dr von Hauner Children’s Hospital, Ludwig Maximilians University, Munich, Germany, and Comprehensive Pneumology Center, Munich (CPC-M), Germany; German Center for Lung Research (DZL), and Institut für Asthma- und Allergieprävention (IAP), Helmholtz Zentrum Munich, Deutsches Forschungszentrum für Gesundheit und Umwelt (GmbH), Munich, Germany, **Gyde Nissen**, University Children’s Hospital, Luebeck, Germany, and Airway Research Center North (ARCN), Germany; German Center for Lung Research (DZL), **Catharina Nitsche**, University Children’s Hospital, Luebeck, Germany, and Airway Research Center North (ARCN), Germany; German Center for Lung Research (DZL), **Frauke Pedersen**, LungenClinic Grosshansdorf GmbH, Grosshansdorf, Germany, and Airway Research Center North (ARCN), Germany; German Center for Lung Research (DZL), **Mareike Price**, Department of Paediatric Pneumology, Allergology and Neonatology, Hannover Medical School, Hannover, Germany, and Biomedical Research in Endstage and Obstructive Lung Disease Hannover (BREATH), Germany; German Center for Lung Research (DZL), **Klaus F. Rabe**, LungenClinic Grosshansdorf GmbH, Grosshansdorf, Germany, and Airway Research Center North (ARCN), Germany; German Center for Lung Research (DZL), **Harald Renz**, Institute of Laboratory Medicine and Pathobiochemistry, Molecular Diagnostics, University of Marburg, Germany, and University of Gießen, Marburg Lung Center (UGMLC); German Center for Lung Research (DZL), **Isabell Ricklefs**, University Children’s Hospital, Luebeck, Germany, and Airway Research Center North (ARCN), Germany; German Center for Lung Research (DZL), **Ernst Rietschel**, University of Cologne, Faculty of Medicine and University Hospital Cologne, Department of Pediatrics, Cologne, Germany, **Barbara Roesler**, Department of Paediatric Allergology, Dr von Hauner Children’s Hospital, Ludwig Maximilians University, Munich, Germany, and Comprehensive Pneumology Center, Munich (CPC-M), Germany; German Center for Lung Research (DZL), **Bianca Schaub**, Department of Paediatric Allergology, Dr von Hauner Children’s Hospital, Ludwig Maximilians University, Munich, Germany, and Comprehensive Pneumology Center, Munich (CPC-M), Germany; German Center for Lung Research (DZL), **Christina Schauberger**, Department of Paediatric Allergology, Dr von Hauner Children’s Hospital, Ludwig Maximilians University, Munich, Germany, and Comprehensive Pneumology Center, Munich (CPC-M), Germany; German Center for Lung Research (DZL), **Tom Schildberg**, University of Cologne, Faculty of Medicine and University Hospital Cologne, Department of Pediatrics, Cologne, Germany, **Carsten Schmidt-Weber**, Center of Allergy and Environment (ZAUM), Technical University of Munich and Helmholtz Center Munich, German Research Center for Environmental Health, Munich, Germany; German Center for Lung Research (DZL), Munich, Germany, **Nicolaus Schwerk**, Department of Paediatric Pneumology, Allergology and Neonatology, Hannover Medical School, Hannover, Germany, and Biomedical Research in Endstage and Obstructive Lung Disease Hannover (BREATH), Germany; German Center for Lung Research (DZL), **Chrysanthi Skevaki**, Institute of Laboratory Medicine and Pathobiochemistry, Molecular Diagnostics, University of Marburg, Germany, and University of Gießen, Marburg Lung Center (UGMLC); German Center for Lung Research (DZL), **Alena Steinmetz**, University Children’s Hospital, Luebeck, Germany, and Airway Research Center North (ARCN), Germany; German Center for Lung Research (DZL), **Laila Sultansei**, University Children’s Hospital, Luebeck, Germany, and Airway Research Center North (ARCN), Germany; German Center for Lung Research (DZL), **Marlen Szewczyk**, LungenClinic Grosshansdorf GmbH, Grosshansdorf, Germany, and Airway Research Center North (ARCN), Germany; German Center for Lung Research (DZL), **Dominik Thiele**, Institute for Medical Biometry and Statistics, University Luebeck, University Medical Centre Schleswig-Holstein, Campus Luebeck, Germany, and Airway Research Center North (ARCN), Germany; German Center for Lung Research (DZL), **Vera Veith**, LungenClinic Grosshansdorf GmbH, Grosshansdorf, Germany, and Airway Research Center North (ARCN), Germany; German Center for Lung Research (DZL), **Gesche Voigt**, University Children’s Hospital, Luebeck, Germany, and Airway Research Center North (ARCN), Germany; German Center for Lung Research (DZL), **Benjamin Waschki**, LungenClinic Grosshansdorf GmbH, Grosshansdorf, Germany, and Airway Research Center North (ARCN), Germany; German Center for Lung Research (DZL), **Henrik Watz**, Pulmonary Research Institute at LungenClinic Grosshansdorf, Grosshansdorf, Germany, and Airway Research Center North (ARCN), Germany; German Center for Lung Research (DZL), **Stefanie Weber**, University Children’s Hospital Marburg, University of Marburg, Germany, and University of Giessen Marburg Lung Center (UGMLC); Member of the German Center for Lung Research, **Markus Weckmann**, University Children’s Hospital, Luebeck, Germany, and Airway Research Center North (ARCN), Germany; German Center for Lung Research (DZL), **Nils Welchering**, Department of Paediatric Allergology, Dr von Hauner Children’s Hospital, Ludwig Maximilians University, Munich, Germany, and Comprehensive Pneumology Center, Munich (CPC-M), Germany; German Center for Lung Research (DZL), **Esther Zeitlmann**, Department of Paediatric Allergology, Dr von Hauner Children’s Hospital, Ludwig Maximilians University, Munich, Germany, and Comprehensive Pneumology Center, Munich (CPC-M), Germany; German Center for Lung Research (DZL), **Ulrich Zissler**, Center of Allergy and Environment (ZAUM), Technical University of Munich and Helmholtz Center Munich, German Research Center for Environmental Health, Munich, Germany; German Center for Lung Research (DZL), Munich, Germany

## Data Availability

The raw data supporting the conclusions of this article will be made available by the authors, without undue reservation.

## Footnotes

1
https://github.com/cytomining/cytominer

2
https://github.com/cells2numbers/unet4neutrophils

3
https://github.com/cells2numbers/migrationminer
