# Supplementary material for: In vitro neutrophil migration is associated with inhaled corticosteroid treatment and serum cytokines in pediatric asthma
Source: Front Pharmacol. 2022 Oct 11;13:1021317. doi: 10.3389/fphar.2022.1021317 (PMC9593213; doi:10.3389/fphar.2022.1021317)
Supplement: Supplementary file 1 [file DataSheet1.docx]

**Appendix A**

| Donor population AN / AT1 / AT2 / C | | | | | |
| --- | --- | --- | --- | --- | --- |
|  | | Asthmatic  (n=75) | | | Control  (n=13) |
| Demographics | | AN (n=30) | AT1 (n=36) | AT2 (n=9) | C |
| Gender | N male (%) | 19 (63.3%) | 21 (58.3%) | 5 (55.6%) | 5 (38.5%) |
| Age | Mean ± SD | 12.3 (±3.9) | 12.7 (±3.8) | 13.5 (±4.2) | 13.1 (±3.4) |
| BMI | Mean ± SD | 21.9 (±7.6) | 21.6 (±6.1) | 21.1 (±4.7) | 19.9 (±3.4) |
| Reported atopy | N atopic (%) | 18 (60%) | 27 (75%) | 6 (66.7%) | 3 (23.1%) |
| Lung function - extended | |  |  |  |  |
| Exh. NO | Mean ± SD | 16.8 (± 20) | 16.4 (±18.3) | 21 (±15.4) | 9.8 (±11.3) |
| FEV1 | Mean ± SD | 105.9 (±14.6) | 103.2 (±13.2) | 101.1 (±9.6) | 106.6 (±13.4) |
| MEF 75 | Mean ± SD | 95 (±20) | 97.2 (±25.7) | 94.4 (±18.4) | 105 (±14.8) |
| MEF 50 | Mean ± SD | 91.1 (±24.4) | 88.7 (±27.9) | 88.7 (±24) | 104.5 (±18.6) |
| MEF 25 | Mean ± SD | 90.4 (±32.6) | 80.1 (±28.8) | 85.8 (±31.2) | 102.9 (±32.6) |
| SR | Mean ± SD | 167.7 (±85.4) | 148.4 (±54.8) | 127.1 (±57.3) | 99.4 (±32.6) |
| RV | Mean ± SD | 96.8 (±38.7) | 116.5 (±73.3) | 95.9 (±31.3) | 123.6 (±47.4) |
| VC | Mean ± SD | 112.5 (±19.3) | 109.7 (±17.7) | 106.3 (±11.4) | 107.9 (±10.4) |
| TLC | Mean ± SD | 109.7 (±13.9) | 112.2 (±21.8) | 103.6 (±5.5) | 111.4 (±12.7) |
| Tiffaneau Index | Mean ± SD | 100.5 (±10.6) | 99.8 (±14) | 97.6 (±6.8) | 103 (±10.5) |
| Blood count | |  |  |  |  |
| Leukocytes (abs.) | Mean ± SD | 7.1 (±1.2) | 7.4 (±1.5) | 8 (±1.6) | 6.7 (±1.5) |
| Segm. Granulocytes (%) | Mean ± SD | 43.5 (±9.8) | 43.3 (±10.3) | 41.7 (±9.6) | 45.5 (±14.8) |
| Eosinophils (%) | Mean ± SD | 4.2 (±2.2) | 5.4 (±4) | 3.3 (±1.9) | 3.5 (±3) |
| Cytokine profiles (CP) | |  |  |  |  |
| Th1 | n high (%) | 2 (6.7%) | 5 (13.9%) | 0 | 0 |
| Th2 | n high (%) | 4 (13.3%) | 6 (16.7%) | 1 (11.1%) | 0 |
| Th9 | n high (%) | 3 (10%) | 3 (8.3%) | 0 | 0 |
| Th17 | n high (%) | 3 (10%) | 4 (11.1%) | 0 | 0 |
| Asthma control | |  |  |  |  |
| good |  | 22 (84.6%) | 11 (36.7%) | 3 (33.3%) | ***AN vs. AT1, ** AN vs. AT2 |

**Table A1. Characterization of AT2 in comparison to AT1. AN and C cluster displayed in Figure 3**. While AN and AT1 present a consistent picture, it becomes apparent that donors of the AT2 cluster (n=5) have increased vital capacity, decreased residual lung volume and a decreased total lung capacity. Some donors belonging to the cluster AN or AT1 present elevated Th1, Th2, Th9 or Th17 cytokine profiles. Though, this is not observable for the AT2 cluster.

| Subpopulation Th1 profiles | | | | |
| --- | --- | --- | --- | --- |
| Demographics | | Th1-low  (n=30) | Th1-high  (n=7) | Level of significance |
| Gender | n male (%) | 16 (53.3%) | 4 (57%) | n.s. |
| Age | Mean (± SD) | 12 (± 3.9) | 10.6 (± 3.9) | n.s. |
| BMI | Mean (± SD) | 20.9 (± 5.4) | 16.7 (± 1) | *** |
| Reported atopy | n atopic (%) | 21 (70%) | 3 (42.9%) | n.s. |
| Steroid treatment | n treated (%) | 20 (66.7%) | 5 (71.4%) | n.s. |
| Lung function | |  |  |  |
| Exhaled NO  ° 1 value missing | Mean (± SD) | 22.1 (± 23.1) | 15.6 (± 17.4)° | n.s. |
| FEV1 | Mean (± SD) | 102.5 (± 13.2) | 103.5 (± 12.1) | n.s. |
| Tiffaneau-Index  # 1 value missing | Mean (± SD) | 98.9 (± 8.4) # | 105.4 (± 9.1) | n.s. |
| Blood count | |  |  |  |
| Leukocytes (abs.) | Mean (± SD) | 7.7 (± 1.5) | 7.2 (± 1.1) | n.s. |
| Segmented granulocytes (%) | Mean (± SD) | 44.9 (± 9.1) | 41.6 (± 11.7) | n.s. |
| Eosinophils (%)  # 1 value missing | Mean (± SD) | 5.2 (± 3.7) # | 5.4 (± 2.9) | n.s. |
| Cytokine profiles | |  |  |  |
| Th2 | n high (%) | 5 (16.7%) | 3 (42.9%) | n.s. |

**Table A2. Demographics of the Th1-high/low phenotype.** Th1-high donors (n=9) present a significantly reduced BMI and are younger and less often atopic (no significance) compared to Th1-low (n=60). In general, the Th1-high group consists of more severe asthma phenotypes that are in need of ICS treatment. Furthermore, Th1-high donors present elevated level of exhaled NO (no significance). Both groups present similar blood counts and lung function.

| Donor population | chemokine | | |
| --- | --- | --- | --- |
|  | fMLP | IL8 | LTB4 |
| Asthma naïve | 21 | 21 | 19 |
| Asthma treated | 34 | 34 | 26 |
| Control | 10 | 12 | 8 |

**Table A3:** The analyzed population included 75 asthma patients and 13 healthy donors (including 30/46 naive/treated asthmatics). Due to rigorous quality control some time lapse experiments were removed and the number of analyzed SiMA experiments varies between chemokines.

| Antibody | Conjugation | Clone | Company | Dilution |
| --- | --- | --- | --- | --- |
| FPR1 | PE | W15086B | Biolegend | 1:10 |
| CD62L | PE | DREG-56 | Biolegend | 1:10 |
| CD184 | PE | 12G5 | BD | 1:10 |
| CD11b | PE | ICRF44 | BD | 1:30 |
| CD16 | PE | 3G8 | BD | 1:100 |
| BLT1 | PE | LTB4R | LsBio | 1:10 |

**Table A4**. Overview antibodies used to analyze surface receptors. All antibodies were used in conjunction with the Zellkraftwerk Cell Safe CytoChip.


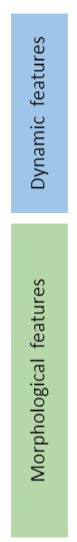


**Feature name**

1 Track_Angle

2 Track_Directionality

3 Track_Speed

4 Track_yFMI

5 Track_xFMI

6 Track_CI

7 Track_MSD

8 Track_Speed_X

9 Track_Speed_Y

10 Track_Speed_max

11 RadialDistribution_FracAtD_brightfield_corrected_1of4

12 AreaShape_Zernike_9_1

13 AreaShape_Zernike_8_4

14 RadialDistribution_FracAtD_brightfield_corrected_2of4

15 AreaShape_Zernike_9_9

16 AreaShape_Zernike_9_7

17 AreaShape_Zernike_9_5

18 AreaShape_Zernike_9_3

19 AreaShape_Zernike_0_0

20 AreaShape_Extent

21 RadialDistribution_MeanFrac_brightfield_corrected_1of4

22 Intensity_StdIntensity_brightfield_corrected

23 AreaShape_FormFactor

24 RadialDistribution_MeanFrac_brightfield_corrected_2of4

25 Intensity_MeanIntensity_brightfield_corrected

**Table A5**: Input features used to calculate the SiMA landscape. Morphological features were created using CellProfiler 3 and migration features were created using MigrationmineR. Data aggregation from single cell features to experiment level profiles were performed using cytominer.


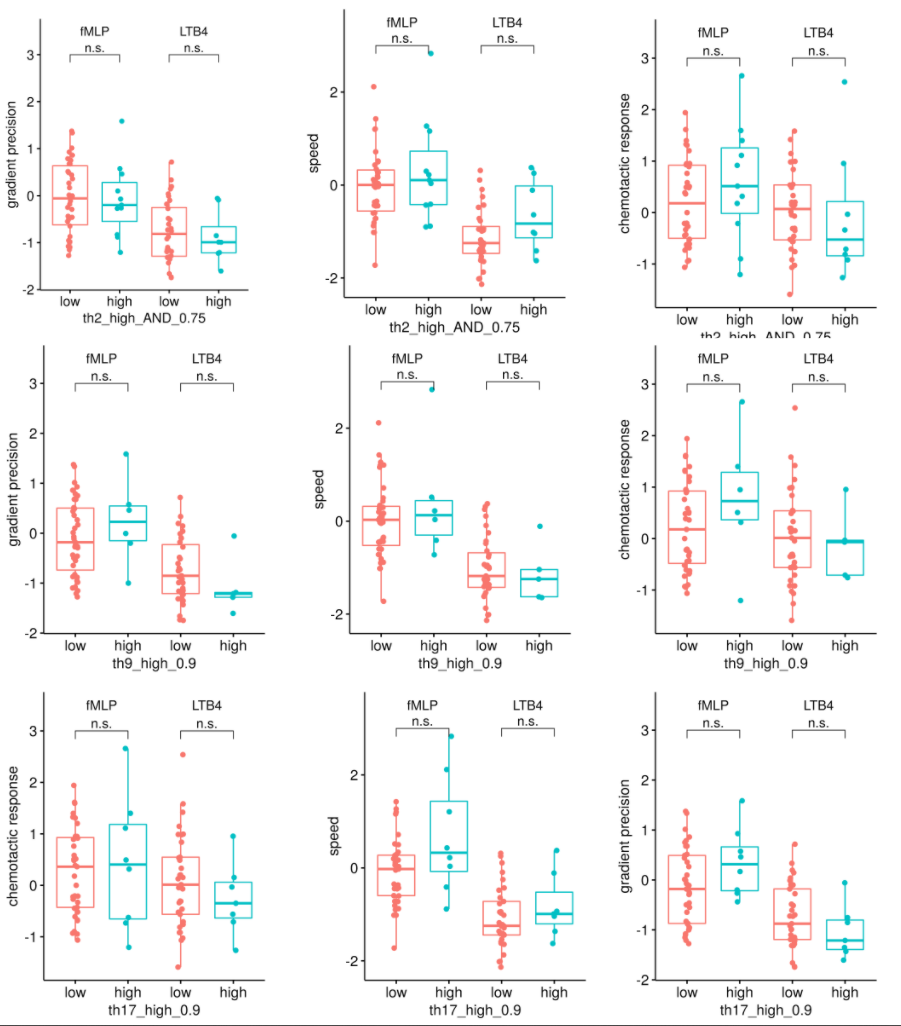


**Figure A1. Migration profiles for TH2, TH9 and TH17 profiles.** TH2 high is defined as patients with cytokine levels for interleukin 4 (IL.4) and interleukin 13 (IL.13) > 75% quantile of all IL.4/IL.13 readouts. Th9 and Th17 are defined as all patients with a interleukin 9 (IL.9) or interleukin g 17 (IL.17) level > 90% quantile of the distribution of IL.9/IL.17 levels. No significant differences could be found for speed, chemotactic response or gradient precision(t-test with significance threshold of p<0.05).
